# Small Nucleolar RNAs: Insight Into Their Function in Cancer

**DOI:** 10.3389/fonc.2019.00587

**Published:** 2019-07-09

**Authors:** Junnan Liang, Jingyuan Wen, Zhao Huang, Xiao-ping Chen, Bi-xiang Zhang, Liang Chu

**Affiliations:** Hepatic Surgery Center, Tongji Hospital, Tongji Medical College, Huazhong University of Science and Technology, Wuhan, China

**Keywords:** small nucleolar RNA, cancer, rRNA processing, mRNA splicing, sdRNA, biomarker, therapy

## Abstract

Small nucleolar RNAs (SnoRNAs) are a class of non-coding RNAs divided into two classes: C/D box snoRNAs and H/ACA box snoRNAs. The canonical function of C/D box and H/ACA box snoRNAs are 2'-*O*-ribose methylation and pseudouridylation of ribosomal RNAs (rRNAs), respectively. Emerging evidence has demonstrated that snoRNAs are involved in various physiological and pathological cellular processes. Mutations and aberrant expression of snoRNAs have been reported in cell transformation, tumorigenesis, and metastasis, indicating that snoRNAs may serve as biomarkers and/or therapeutic targets of cancer. Hence, further study of the functions and underlying mechanism of snoRNAs is valuable. In this review, we summarize the biogenesis and functions of snoRNAs, as well as the association of snoRNAs in different types of cancers and their potential roles in cancer diagnosis and therapy.

## Introduction

Small nucleolar RNAs (snoRNAs) are an extensively studied non-coding RNAs that primarily accumulate in the nucleoli and consist of 60–300 nucleotides (NTs). SnoRNAs are mostly responsible for the posttranscriptional modification and maturation of ribosomal RNAs (rRNAs), small nuclear RNAs (snRNAs), and other cellular RNAs. SnoRNAs are divided into two classes: C/D box snoRNAs and H/ACA box snoRNAs. C/D box snoRNAs guide−2′-*O*-ribose methylation, and H/ACA box snoRNAs direct the pseudouridylation of NTs (1–4).

**Figure 1:**
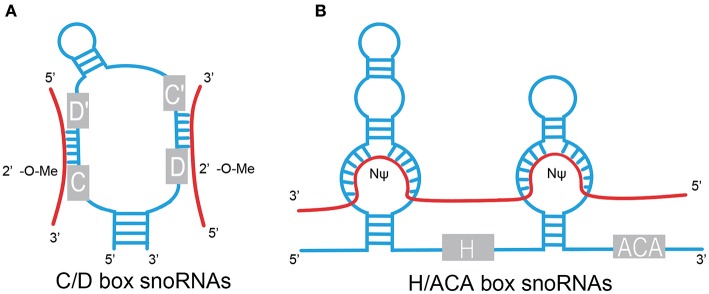
Structural features of C/D box and H/ACA box snoRNAs. **(A)** C/D box. snoRNAs contain two conserved sequence elements, named box C (RUGAUGA), and box D (CUGA). Box C and box D are close to each other by the base pairing of the 5′ and 3′ termini and fold into a k-turn motif. The antisense elements, upstream of the box D/D′ motifs, are complementary to target RNAs and catalyze site-specific 2'-O-methylation (2′-O-Me) of the NT in target RNAs. **(B)** H/ACA box snoRNAs contain evolutionarily conserved structural elements, including box H (ANANNA), box ACA motif, and two pseudouridylation pockets. Pseudouridylation pockets are complementary to the substrate RNAs and responsible for pseudouridylation (NΨ). Red line, target RNAs.

The box C/D family of snoRNAs is characterized by a kink-turn (k-turn) (stem–bulge–stem) structure and contains two conserved sequence elements: box C (RUGAUGA) and box D (CUGA) located at the 5′ and 3′ ends of the RNA molecule, respectively (Figure 1) (1). The k-turn motif is essential to the assembly of a small nucleolar ribonucleoprotein (snoRNP) including fibrillarin, Nop56, Nop58, and 15.5kD (3, 5, 6). Fibrillarin is an enzyme that is responsible for substrate methylation. Nop56, Nop58, and 15.5kD contribute to maturation, stability, and localization of snoRNAs. There are a few C/D box snoRNAs that do not form canonical snoRNPs to function in RNA methylation (7, 8). Most C/D box snoRNAs contain less conserved copies of box C and box D in the central region of the snoRNA, identified as box C' and box D', respectively, which generally carry one or two base modifications. The upstream elements of the box D/D′ motifs are complementary to target RNAs, allowing for alignment and methylation of appropriate NTs (3, 9, 10).

H/ACA box snoRNAs are composed of conserved box H and box ACA motifs (Figure 1). Box H is an ANANNA sequence (N represents any NT), and ACA box is a trinucleotide (ACA). H/ACA box snoRNAs are marked by the “hairpin–hinge–hairpin–tail” secondary structure with box H in the hinge region and box ACA on the 3′ end of the last hairpin. An internal loop is located in the hairpin of H/ACA box snoRNAs with a 9–13 NT sequence on each strand complementary to the substrate RNAs, which forms a so-called pseudouridylation pocket. The distance between the site responsible for pseudouridylation and the H/ACA box is 14–16 NTs (3, 6, 9, 11). Similar to the C/D box snoRNAs, a set of core proteins are correlated with the H/ACA box snoRNAs to form stable and functional snoRNPs, including NHP2, NOP10, GAR1, and the pseudouridine synthase dyskerin (8).

## The Biogenesis of snoRNAs

In vertebrates, most snoRNAs are encoded in the introns of protein-coding or non-coding genes, except for a small number of snoRNAs transcribed autonomously by RNA polymerase II (3). The biogenesis of most intronic snoRNAs includes cotranscription with the host gene, splicing, debranching of the intron lariat, and exonucleolytic digestion in the nucleoplasm. Recruitment of ribonucleoproteins to the nascent intronic snoRNAs is essential to the maturation of snoRNAs; this process is cotranscriptionally initiated. These proteins are critical to both processing stability and nucleolar localization (3, 12). In addition, many auxiliary factors are involved in snoRNP assembly and maturation, such as Shq1, Naf1, and NUFLP. SnoRNPs are transported to Cajal bodies, where they perform additional processing and maturation tasks (13). Thereafter, they are delivered to the nucleolus.

## The Functions of snoRNAs

### Participation in rRNA Processing

One well-studied function of snoRNAs is their role in the modification, maturation, and stabilization of rRNA (3). As the research intensifies, hundreds of 2′-*O*-methylation or pseudouridylation residues located within conserved and functional regions of rRNAs have been found. A sequence upstream of box D and/or box D' recognizes target RNAs and results in the 2′-*O*-methylation of the fifth NT by the methylase fibrillarin (7, 14, 15). The conversion of uridines to pseudouridine is carried out by dyskerin, and the site destined for pseudouridylation is located 14–16 NTs upstream of box H and/or box ACA (9, 16, 17).

In addition to the rRNA modification function, some snoRNAs act in on pre-rRNA cleavage (18, 19). For example, SNORD3 promotes the proper pre-rRNA formation for subsequent endonucleotic processing (20–23). Furthermore, SNORD118, SNORD14, SNORD22, SNORA71, and probably, SNORD13 are involved in pre-rRNA cleavage (24–27).

### Regulation of mRNA Splicing and Editing

Recent evidence indicates that snoRNAs play an important role in the regulation of gene expression. SNORD115 is encoded in the imprinted SNURF–SNRPN locus on human chromosome 15, which is frequently deleted in Prader–Willi syndrome (PWS) (28–30). SNORD115 contains an 18-NT sequence complementary to the alternative exon Vb of the serotonin receptor subtype 2C (5-HT2cR) and regulates the alternative splicing of 5-HT2cR (31). Besides the regulation of alternative splicing, SNORD115 influences RNA editing. The site-specific adenosine-to-inosine (A-to-I) base conversion is essential to the generation of 5-HT2cR (32). Vitali et al. demonstrated that SNORD115 inhibited the efficiency of the ADAR2-mediated RNA editing of 5-HT2cR by forming a bona fide snoRNP particle (33). Moreover, using bioinformatic prediction and experimental verification, Rishore et al. identified that SNORD115 regulated the alternative splicing of five pre-mRNAs (DPM2, TAF1, RALGPS1, PBRM1, and CRHR1). They demonstrated that SNORD115 generated shorter RNAs, called processed small nucleolar RNAs (psnoRNAs), that directed the regulation of alternative splicing (34). Two gene clusters encoding SNORD115 and SNORD116 are located in the imprinted locus 15q11-q13 containing 47 repeats of SNORD115 along with 27 copies of SNORD116. Using genome-wide array analysis after overexpressing SNORD115 and SNORD116 in human embryonic kidney (HEK) 293T cells, Falaleeva et al. found that SNORD115 and SNORD116 influenced the expression levels of over 200 genes and modified each other's activity (35). Bazeley et al. found that energetically favorable putative targets of SNORD116 were correlated with exons that were capable of alternative splicing and speculated that SNORD116 was involved in the regulation of alternative splicing (36). Wu et al. identified that 5′ snoRNA-capped and 3′ polyadenylated lncRNA (SPA) required snoRNP complexes to protect them from trimming by 5′-3′ exoribonuclease 2 (XRN2). The most well-understood function of SNORD27 is to guide the methylation of rRNA. Recent research demonstrated that SNORD27 regulated the alternative splicing of the transcription factor E2F7 pre-mRNA by directing RNA–RNA interaction (7). A similar function was reported for SNORD88C, which produced small RNAs derived from snoRNAs (sdRNAs) containing the box C' that was complementary to several pre-mRNAs including FGFR3 and regulated the alternative splicing of FGFR3 pre-mRNA (37). In another study, Huang et al. discovered that SNORA50A inhibited mRNA 3' processing by blocking the Fib1-poly(A) site interaction, which was the first report that snoRNA regulated mRNA 3' processing (38).

### Involvement in Stress Response and Metabolic Homeostasis

Michel et al. found that three snoRNAs (SNORD32A, 33, and 35A) encoded in the ribosomal protein L13a (Rpl13a) locus were increased significantly under oxidative stress induced by palmitate and hydrogen peroxide treatment. Knockdown of these three snoRNAs simultaneously increased resistance against palmitate *in vivo* (39). Furthermore, snoRNA ACA11 has been found to suppress oxidative stress by downregulating ribosomal protein genes and other snoRNAs (40). Brandis et al. identified that the loss of snoRNA U60 reduced plasma membrane-to-endoplasmic reticulum cholesterol trafficking and increased *de novo* cholesterol synthesis. This finding suggested that U60 played a function in regulating intracellular cholesterol trafficking (41). Other reports demonstrated that snoRNAs regulate cellular metabolic homeostasis. For example, snoRNA U17 regulated cellular cholesterol trafficking *via* the encoding of hypoxia-upregulated mitochondrial movement regulator (HUMMR) by its target mRNA. Four snoRNAs encoded in Rpl13a, snoRNAs U32A, U33, U34A, and U35A, regulated systemic glucose metabolism (42, 43).

### The Role and Molecular Mechanisms of snoRNAs in Cancer

SnoRNAs are widely involved in many physiological and pathological processes; indeed, emerging evidence suggests that snoRNAs have tumor-suppressive or oncogenic functions in various cancer types (Table 1). SnoRNAs are reported to participate in many biological cancer processes, including the invasion of growth suppressors and cell death, activation of invasion and metastasis, and angiogenesis and sustained proliferative signaling. The underlying molecular mechanisms are diverse.

**Table 1:** SnoRNAs involved in human cancers.

**snoRNAs**	**Class**	**Expression level**	**Function**	**Cancer**	**Intersection molecules and/or pathway**	**Reference**
SONRA42/MBI-43	H/ACA box	Increased	OG	NSCLC, CRC	P53	(44, 45)
SNORD78/U78	C/D box	Increased	OG	NSCLC, PCa		(46)
U50/SNORD50A/RNU50	C/D box	Decreased	TS	BRCA, PCa	Ras-ERK1/ERK2	(47–49)
U3/SNORD3@	C/D box	Increased	OG	BRCA	p53	(50)
U8/SNORD118/LCC	C/D box	Increased	OG	BRCA	p53	(50)
RNU44/SNORD44/U44	C/D box	Decreased	TS	BRCA		(51)
RNU43/SNORD43/U43	C/D box	Decreased	TS	BRCA		(51)
SNORD48/U48/RNU48	C/D box	Decreased	TS	BRCA		(51, 52)
SNORD29/U29/RNU29	C/D box		OG	BRCA	YB-1	(52)
SNORD34/U34/RNU34	C/D box		OG	BRCA	YB-1	(52)
SNORD67/HBII-166	C/D box		OG	BRCA	YB-1	(52)
SNORD33/U33/RNU33	C/D box		OG	BRCA	YB-1	(52)
ACA44/SNORA44	H/ACA box		OG	BRCA	YB-1	(52)
SNORD114-1/14q(II-1)	C/D box	Increased	OG	APL	Rb/p16	(53)
SNORD112-114	C/D box	Increased	OG	APL		(53)
SNORD35A/RNU35/RNU35A/U35	C/D box	Increased	OG	AML	AES and DDX21	(54)
SNORD74/U74/Z18	C/D box	Increased	OG	AML	AES and DDX21	(54)
SNORD14D	C/D box	Increased	OG	AML	AES and DDX21	(54)
SNORD43/RNU43/U43	C/D box	Increased	OG	AML	AES and DDX21	(54)
SNORA21/ACA21	H/ACA box	Increased	OG	CRC		(55)
SNORA15/ACA15A	H/ACA box	Increased	OG	CRC		(56)
SNORA41/ACA41A	H/ACA box	Increased	OG	CRC		(56)
SNORD33/U33/RNU33	C/D box	Decreased	TS	CRC		(56)
SNORA18L5	H/ACA box	Increased	OG	HCC	stress-RPs-MDM2-P53	(57)
SNORD126/MIR1201/MIRN1201	C/D box	Increased	OG	HCC	PI3K–AKT	(58)
ACA11/SCARNA22	H/ACA box	Increased	OG	HCC, MM	PI3K–AKT, RP genes	(40, 59)
SNORD76/U76	C/D box	Increased/Decreased	OG/TS	HCC,GBM	Wnt/-catenin pathway	(60, 61)
SNORA47/HBI-115	H/ACA box	Increased	OG	HCC		(62)
SNORD113-1/14q(I-1)	C/D box	Decreased	TS	HCC	ERK1/2 and SMAD2/3	(63)
SNORA55/ACA55	H/ACA box	Increased	OG	PCa		(64)
SNORD59A/U59/RNU59	C/D box	Decreased	TS	PCa		(65)
SNORD82/RNU82/U82/Z25	C/D box	Decreased	TS	PCa		(65)
SNORD116/HBII-85	C/D box	Decreased	TS	PCa		(65)
SNORD117/U83	C/D box	Decreased	TS	PCa		(65)
SNORD24/RNU24/U24	C/D box	Decreased	OG	ACC	miRNAs	(66)
SNORA74B/U19-2	H/ACA box	Decreased	TS	GBC	AKT/mTOR	(67)
SNORD47/RNU47/U47	C/D box	Decreased	TS	GBM		(68)
SNORD94/U94	C/D box	Increased	OG	Osteosarcomas	Mutant p53, Est2	(69)
SNORA70/DXS648E/RNU70/U70	H/ACA box	Increased	OG	Osteosarcomas	Mutant p53, Est2	(69)
SNORD10/mgU6-77	C/D box	Increased	OG	Osteosarcomas	Mutant p53, Est2	(69)
SNORA13/ACA13	H/ACA box	Increased	OG	Osteosarcomas	Mutant p53, Est2	(69)
SNORA38/ACA38	H/ACA box	Increased	OG	Osteosarcomas	Mutant p53, Est2	(69)
SNORA79/ACA65A	H/ACA box	Increased	OG	Osteosarcomas	Mutant p53, Est2	(69)
SNORA46/ACA46	H/ACA box	Increased	OG	Osteosarcomas	Mutant p53, Est2	(69)
SCARNA9/Z32/mgU2-19/30	SCARNA	Increased	OG	Osteosarcomas	Mutant p53, Est2	(69)
SNORA23/ACA23	H/ACA box	Increased	OG	PDAC	SYNE2	(70)
SNORD35B/RNU35B/U35B	C/D box	Decreased	TS	HNSCC		(71)
SNORD114-10/14q(II-10)	C/D box	Decreased	TS	OC		(72)

*OG, oncogene; TS, tumor suppressor; BRCA, breast cancer; PCa, prostate cancer; APL, acute promyelocytic leukemia; MM, multiple myeloma; GBM, glioblastoma; ACC, adenocarcinoma; GBC, gallbladder cancer; PDAC, pancreatic ductal adenocarcinoma; HNSCC, head and neck squamous cell carcinoma; OC, ovarian cancer*.

### SnoRNAs and the P53 Regulatory Pathway in Cancer

P53 is a well-known tumor suppressor that responds to diverse cellular stresses to regulate the expression of target genes involved in cell cycle arrest, apoptosis, and DNA repair (73). Increasing evidence suggests that snoRNAs are closely associated with the p53 regulatory pathway. For instance, a recent study identified that snoRNAs and FBL were usually overexpressed in human breast and prostate cancers and that this overexpression promoted tumorigenicity *in vitro* and *in vivo* (74). Further research found that oncogene Myc upregulated the expression level of FBL and led to elevated snoRNA biogenesis, inducing p53 suppression. Cellular stress induced by knockdown of snoRNA pathway genes increased the accumulation of p53 by promoting the binding of ribosomal protein L5 (RPL5) and ribosomal protein L11 (RPL11) with MDM2 and led to p53 stabilization. Furthermore, impaired snoRNA biogenesis induced by FBL depletion promoted PTB binding to the p53 internal ribosome entering site (IRES) and regulated the initiation of p53 translation (74). A similar finding has been reported by Langhendries et al. U3 and U8 were upregulated in breast cancer, and depletion of U3 and U8 inhibited tumorigenicity of breast cancer cells *in vivo* and *in vitro* (50). Depletion of U3 or U8 led to ribosome biogenesis dysfunction by inhibiting pre-rRNA processing and reduced production of mature rRNAs (50). SnoRNA42 was overexpressed in non-small cell lung cancer (NSCLC) and played an oncogenic role by regulating the expression of p53. Doxorubicin-induced DNA damage induced the expression of the GAS5-derived snoRNAs (U44 and U77) in a p53-dependent manner. Chromatin immunoprecipitation sequencing (ChIP-seq) experiments confirmed that p53 directly controlled GAS5 transcription. GAS5-derived snoRNAs are closely correlated with p53 levels in colorectal tissue, suggesting that GAS5-derived snoRNAs have a critical role in p53-associated signaling pathways in colorectal cancer (CRC) (75).

There are several other snoRNAs associated with the p53 regulatory pathway. In a multistage germline copy number variation (CNV)-based genome-wide association studies (GWAS), a low-frequency duplication at 15q13.3 containing SNORA18L5 was strongly correlated to risk of hepatitis B virus (HBV)-related hepatocellular carcinoma (HCC). Further studies showed that SNORA18L5 was overexpressed in HCC tissues compared with adjacent normal tissues and played an oncogenic role in HCC (57). Overexpression of snoRNA18L5 kept RPL5 and RPL11 from binding to MDM2, resulting in increased MDM2-mediated ubiquitination degradation of p53 (57). It is well known that p53 mutations in cancers often exert oncogenic gain-of-function properties that contribute to tumorigenicity. Increasing evidence shows that in addition to wild-type p53, mutant p53 also interacts with snoRNAs in cancers (76). For instance, Pourebrahim et al. identified that p53-mutant mice developed osteosarcomas with increased metastasis as compared with p53-null mice. Using comprehensive transcriptome RNA sequencing analysis of 16 tumors, they also found that a cluster of snoRNAs were upregulated in p53 mutant tumors. An analysis of regulatory elements showed that the Est2-binding motif was highly enriched in these deregulated snoRNAs. Genetic deletion of the Esr2 transcription factor reduced the expression of these snoRNAs in p53 mutant mice and abrogated the mutant p53 prometastatic phenotype (69).

### SnoRNAs in the Regulation of Other Cancer-Related Signaling Pathways

There are many other signaling pathways associated with snoRNAs, including the phosphoinositide 3-kinase (PI3K)–AKT and Wnt/β-catenin pathways discussed here. The PI3K–AKT pathway is a highly conserved cell signaling system in most multicellular organisms, which is critical to diverse cellular processes such as stemness, cell proliferation, differentiation, and cell death (77). The role of the PI3K–AKT signaling pathway in cancer is well-documented (77). A number of snoRNAs have been implicated in PI3K–AKT signaling, either directly or indirectly. ACA11 knockdown significantly decreased the phosphorylation of PI3K and AKT, whereas ACA11 overexpression promoted HCC cell growth and metastasis by activating the PI3K–AKT pathway (59). In another study, SNORD126 activated PI3K–AKT signaling by upregulating FGFR2 and promoted HCC and CRC cell growth (58). PH domain leucine-rich repeat protein phosphatase (PHLPP) is an endogenous inhibitor of the AKT pathway (77). In gallbladder cancer, SNORA74B knockdown suppressed activation of the AKT/mechanistic target of rapamycin (mTOR) pathway *via* inducing PHLPP expression (67). In the Wnt/β-catenin pathway, the major pathway regulating the development and progression of HCC, SNORD76 knockdown significantly decreased the level of β-catenin, c-Myc, and cyclinD1. Conversely, overexpression of SNORD76 promoted HCC tumorigenicity through activation of the Wnt/β-catenin pathway (60).

### SnoRNAs as the Precursors of Small RNAs

In 2008, Kawaji et al. performed an unbiased sequencing of human small RNAs (19–40 NTs) and found independent classes of small RNAs originating from noncoding RNAs (78). Since then, a wealth of sdRNAs have been identified. In 2009, Taft et al. performed a systematic analysis of small RNA by deep sequencing libraries from various eukaryotic tissues and found that small RNAs with evolutionarily conserved size and position were derived from a large proportion of snoRNA loci in animals (human, mouse, chicken, and fruit fly), *Arabidopsis*, and fission yeast (79). In recent years, the processing patterns of snoRNAs have raised substantial attention, and increasing evidence shows that these small RNAs have a role in cancers. SnoRNA–miR-28, an miRNA-like non-coding RNA derived from snoRNAs, was significantly upregulated in breast tumors and promoted proliferation of tumor cells (80). SNORD28, a p53-repressed snoRNA located in SNHG1, can be processed into smaller miRNA-like molecules that are capable of binding argonaute (AGO) and exerting miRNA-like effects. This procession can be repressed by p53, the underlying mechanism of which is unclear (80). At the same time, snoRNA–miR-28 acts as an miRNA and directly interacts with TAF9B's 3'-untranslated region (3'-UTR), leading to the reduction of TAF9B mRNA and protein expression levels. The reduced expression impairs the stability of p53 by promoting the binding of MDM2 to p53. Collectively, a regulatory loop exists between p53, SNHG1, snoRNA-miR-28, and TAF9B (80). miR-768-5p, another miRNA-like non-coding RNA derived from SNORD17, was reported to bind to YB-1 and may play a role in the development of cancers (52).

PIWI-interacting RNAs are identified as inhibitor of transposable elements (TEs) in the germline and play an important role in regulating target RNA to silence its expression *via* base-pairing recognition (81). In addition to miRNA-like functions, these sdRNAs also have the ability to play piRNA-like functions. Pi-sno-75, a piRNA derived from SNORD75 located in GAS5, can specifically bind to PIWIL1 and PIWIL4 (piRNA binding proteins in *Homo sapiens*) and bearing modification of 2′-*O*-methylation at the 3′ terminus (82). Microarray results showed that TRAIL, a tumor-specific suppressor, was upregulated by pi-sno-75 synthesized in breast cancer cells. ChIP results showed that overexpression of pi-sno-75 increased the H3K4me3 level and decreased the H3K27me3 level within −169 base pair (bp) of TRAIL promoter in a PIWIL1/PIWIL4-dependent manner. Further research identified the molecular mechanism of this regulation: the pi-sno-75/PIWIL complex can interact with WDR5 and recruit the entire hCOMPASS-like complex along with SMYD3 to the promoter region of the TRAIL gene (82). Pi-sno-75 exhibits antitumor activity *in vitro* and *in vivo* by upregulating the expression of TRAIL (82). Uzunova et al. identified that sdRNAs derived from SNORD44, SNORD74, SNORD78, and SNORD81 were upregulated in prostate cancer. In particular, the levels of, SNORD78 and its sdRNAs were obviously higher in patients with metastatic diseases (65).

### The Role of snoRNAs in Cancer Stem Cells

Cancer stem cells have the capacities of self-renewal, differentiation, and tumorigenicity. The presence of cancer stem cells has been reported in various cancers, which may explain why current chemotherapies cannot consistently eradicate cancers (83). A growing body of research suggests that snoRNAs play an important role in cancer stem cells. Self-renewal activity is essential in leukemogenesis. Zhou et al. identified that amino-terminal enhancer of split (AES), C/D box snoRNAs, and rRNA 2′-*O*-methylation are essential for the AML1–ETO–induced self-renewal of leukemia cells *in vitro* and *in vivo* (54). Knockdown of AES leads to reduction of snoRNAs (primarily C/D box snoRNAs) and suppression of translation efficiency in AE9a-transduced leukemia cells. DDX21 is an RNA helicase that binds to snoRNA/RNP to facilitate rRNA modification. The underlying mechanism involves the knockdown of AES, which reduces the association of DDX21 with the C/D box snoRNP complex including FBL, NOP598, NOP56, and NCL and followed by suppression of snoRNAs. C/D box snoRNAs are highly expressed in acute myeloid leukemia (AML) and correlated closely with the *in vivo* frequency of leukemic stem cells. Knockdown of SNORD14D or SNORD35A suppressed the clonogenic potential of leukemia cells *in vitro* and delayed leukemogenesis *in vivo* (54). In previous studies, Mannoor et al. demonstrated that (aldehyde dehydrogenase 1) was a cancer stem cell marker, as ALDH1^+^ cancer cells have extensive self-renewal, proliferation, and *in vivo* tumorigenic potentials. There are 22 snoRNAs that display differential expression in ALDH^+^ cancer cells compared with ALDH^−^ cancer cells (44). SNORA3 and SNORA43 overexpressed in lung cancer stem cells and inversely associated with survival in NSCLC patients. Along with CD133, another important maker for lung cancer stem cells, SNORA42 was confirmed to be especially dysregulated in lung cancer stem cells (44). Knockdown of SNORA42 inhibited self-renewal capacity and *in vitro* tumorigenesis by inducing apoptosis and reducing the transcript level of stem cell-associated genes including OCT4, Nanog, Sox2, Notch1, Smo, and ABCS2, suggesting SNORA42 may associate with the expression of core stem cell transcription factors in lung cancer stem cells (44). SNORD78 is also reported to be upregulated in cancer stem cells in NSCLC and is required for the self-renewal of cancer stem cells in NSCLC (46).

### SnoRNAs as Biomarkers and Therapeutic Targets of Cancers

As mentioned above, aberration in the expression of snoRNAs was found to be prevalent in many cancers; some of these anomalies are cancer type specific. Substantial research has shown that many snoRNAs are stably expressed and detectable in body fluids including the blood plasma, serum, and urine of cancer patients. Their expression levels are closely associated with diagnosis, prognosis, and classification of subtypes. Given these specifics, snoRNAs have the potential to become cancer biomarkers (84, 85).

The clinical value of snoRNA expression analysis in the diagnosis of certain subtypes of peripheral T-cell lymphoma (PTCL) has been demonstrated. Moreover, Valleron et al. found that the overexpression of snoRNA HBI-239 and HBI-239-processed miRNA predicted good prognosis in angio-immunoblastic T-cell lymphoma (AITL) and PTCL not otherwise specified (PTCL-NOS) (86). Berquet et al. investigated the snoRNA expression profiles in B-cell chronic lymphocytic leukemia (CLL) patients and identified that immunoglobulin heavy chain variable region gene (IGHV)-mutant patients exhibiting overexpression of 20 snoRNAs had a shorter treatment-free survival (TFS) (87). SNORA70F and SNORD116-118 were developed into a 2-snoRNA model that appeared to distinguish two different prognostic CLL groups (88). Furthermore, a research group identified that snoRNA expression profiles can be used for the classification of leukemia subgroups due to the differential expression among these various groups (89). SNORD33, SNORD66, and SNORD76 were overexpressed in NSCLC patients and were detectable in plasma, yielding 81.1% sensitivity and 95.8% specificity in distinguishing NSCLC patients. SNORA42 was overexpressed in NSCLC patients; the levels were inversely correlated with the survival of patients, providing a potential marker for diagnosis and prognosis in NSCLC. SNORA47, SNORA68, and SNORA78 were reported to accurately predict overall survival of NSCLC patients in a training set of 77 cases, which may become an snoRNA-based model for predicting overall survival in lung cancer patients (44, 90, 91). SNORD42 and SNORD21 were also reported to be promising predictive biomarkers for prognosis in CRC patients (55, 92). The expression level of SNORA18L5 in HCC tissues was correlated with the survival time of patients; patients with high SNORA23 expression had a shorter disease-free survival time (57, 70). RUN44, RUN43, and RUN48 were downregulated in breast cancer and were associated with a poor prognosis. In a recent study, Krishnan et al. found a large number of snoRNAs that were promising prognostic markers for breast cancer (51, 93). SNORD114-10 was downregulated in metastatic omentum tissues, suggesting that this snoRNA may provide a prediagnostic biomarker for ovarian cancer metastasis (72). These studies strongly indicate that snoRNAs offer promising novel diagnostic and prognostic markers across a range of cancer types. However, further investigation is necessary, and many challenges must be overcome prior to their application in clinical settings.

Since snoRNAs participate in tumorigenesis, tumor aggressiveness, and staging, they are ideal candidates for cancer therapy. The regulation of snoRNA expression may contribute to the goal of cancer treatment. For example, antisense oligonucleotide (ASO)-mediated downregulation of SNORA23 expression reduced tumor growth, dissemination, and liver metastasis in pancreatic ductal adenocarcinoma (70). Our studies demonstrated that SNORD44 was downregulated in CRC and that overexpression of SNORD44 by an oncolytic adenovirus inhibited CRC growth (94). With the expansion of research and the further development of applicable technologies, snoRNAs may become major cancer therapeutic targets in the near future.

## Conclusions and Prospects for Further Research

Although the landscape of snoRNA function in its entirety remains still unclear, previous studies have confirmed the critical roles of snoRNAs in rRNA processing, gene transcription, RNA splicing, and other cellular processes. As sequencing and microarray analysis technologies evolve, more and more tumor-related snoRNAs are being discovered. Recent studies have demonstrated that aberrant expression and mutations in specific snoRNAs were associated with tumorigenesis and metastasis. The tissue- and cancer-specific expression of snoRNAs can be used as reliable prognostic markers for cancer diagnosis and novel therapeutic targets. However, the field is still in its infancy, and our understanding of the role of snoRNAs in tumors remains incomplete. Currently, the effect of snoRNA on cellular signaling pathways is also poorly understood. Functional screening of RNA interference libraries and/or clustered regularly interspaced short palindromic repeats (CRISPR)-based libraries is necessary to explore snoRNA functions in signaling pathways. SnoRNAs do not always act directly; indeed, most of their actions are indirect binding to other molecules. The identification of more molecules that interact with snoRNAs using RNA precipitation combined with high-throughput mass spectrometry and RNA immunoprecipitation (RIP) technologies is promising. Moreover, animal models are important for understanding the function of non-coding RNA in tumorigenesis and development. However, animal models of snoRNAs in cancer remain insufficient to date. Furthermore, emerging evidence suggests that some non-coding RNAs contain translated open reading frames that are able to encode proteins. This hints at whether snoRNAs also have the ability to encode proteins and provides a whole new direction for investigating snoRNA function. A better grasp of the role of snoRNAs in tumors will help us to understand tumors more comprehensively and may offer not only novel diagnostic biomarkers but also effective therapeutic targets in the near future.

## Author Contributions

All authors listed have made a substantial, direct and intellectual contribution to the work, and approved it for publication.

### Conflict of Interest Statement

The authors declare that the research was conducted in the absence of any commercial or financial relationships that could be construed as a potential conflict of interest.
